# Combinatorial actions of IL-22 and IL-17 drive optimal immunity to oral candidiasis through SPRRs

**DOI:** 10.1371/journal.ppat.1012302

**Published:** 2024-07-01

**Authors:** Felix E. Y. Aggor, Martinna Bertolini, Bianca M. Coleman, Tiffany C. Taylor, Nicole O. Ponde, Sarah L. Gaffen

**Affiliations:** 1 University of Pittsburgh, Division of Rheumatology and Clinical Immunology, Pittsburgh, Pennsylvania, United States of America; 2 University of Pittsburgh, Department of Periodontics and Preventive Dentistry, Pittsburgh, Pennsylvania, United States of America; Rutgers New Jersey Medical School, UNITED STATES

## Abstract

Oropharyngeal candidiasis (OPC) is the most common human fungal infection, arising typically from T cell immune impairments. IL-17 and IL-22 contribute individually to OPC responses, but here we demonstrate that the combined actions of both cytokines are essential for resistance to OPC. Mice lacking IL-17RA and IL-22RA1 exhibited high fungal loads in esophagus- and intestinal tract, severe weight loss, and symptoms of colitis. Ultimately, mice succumbed to infection. Dual loss of IL-17RA and IL-22RA impaired expression of small proline rich proteins (SPRRs), a class of antimicrobial effectors not previously linked to fungal immunity. Sprr2a1 exhibited direct candidacidal activity in vitro, and *Sprr1-3a*^*-/-*^ mice were susceptible to OPC. Thus, cooperative actions of Type 17 cytokines mediate oral mucosal anti-*Candida* defenses and reveal a role for SPRRs.

## Author summary

Oral thrush is a painful oral infection of the mouth. We show that the combined actions of 2 immune cytokines, IL-17 and IL-22, act in a concerted manner to combat this infection, in part via small proline rich proteins.

## Introduction

The commensal pathobiont *Candida albicans* is the most frequent cause of human fungal infections, yet studies of fungal immunity lag considerably behind other microbes [1]. Oropharyngeal candidiasis (OPC) ranges from a nuisance to a severe and painful condition, with potential to cause nutritional deficits or esophageal cancer [2–5]. To date, there are no vaccines to *C*. *albicans* or indeed to any fungi, highlighting a need to better define the correlates of immunity required to restrain fungal pathogenesis [1,6].

The importance of the Th17 axis in OPC was first demonstrated in mice lacking IL-17R subunits (IL-17RA, IL-17RC), subsequently validated in humans lacking IL-17R components or downstream signaling components [7–13]. Nonetheless, OPC occurs infrequently upon clinical IL-17 blockade, implying that additional pathways contribute to disease [14,15]. Th17 cells produce cytokines in addition to IL-17, notably IL-22 [16]. Despite co-expression in similar lymphocyte populations, IL-17 and IL-22 signal via distinct receptors that are located on distinct epithelial populations, with IL-17 acting on superficial stratified squamous epithelial cells and IL-22 acting on stem-like basal cell populations [17,18]. Moreover, these cytokines activate different signaling modalities (TRAF/NF-κB/mRNA stabilization versus JAK-STAT activation) [19]. How these cytokines act coordinately to achieve antifungal immunity is unclear.

We report that mice lacking the IL-17 and IL-22 receptors are exquisitely susceptible to OPC, more than either knockout alone. Infection is associated with up-regulation of small proline-rich (SPRR) proteins, a poorly defined class of antimicrobial effectors [20,21]. We show that SPRRs exert direct candidacidal activity and contribute cooperatively to OPC immunity.

## Results

### IL-17 and IL-22 signals are nonredundant in oral candidiasis

**Fig 1:**
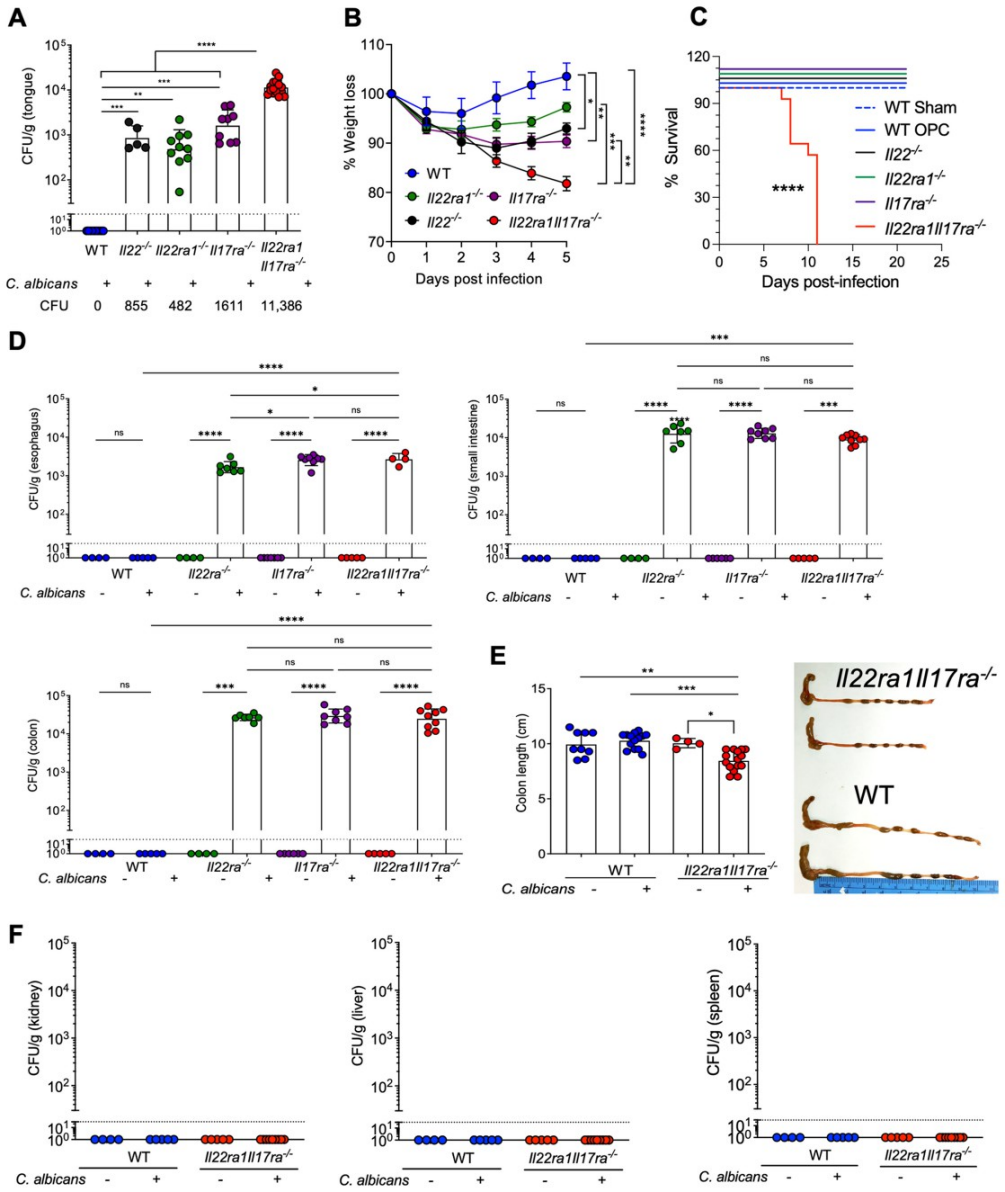
*IL-17* and *IL-22* are nonredundant in OPC. **(A**) Oral fungal loads at day 5. Dashed line: limit of detection. One-way ANOVA with Tukey’s test. **(B)** Weight loss, two-way ANOVA with Tukey’s test. **(C)** Kaplan–Meier survival curve, Mantel–Cox log-rank test. **(D)** Fungal loads in indicated organs at day 5. Data from 3–9 samples/group. **(E)** Left: representative colons. Right: colon length, mean + SEM. Data from 2–3 independent experiments. **(F)** Fungal loads in the indicated organs at day 5.

Here, we assessed the magnitude of OPC in *Il22ra1Il17ra*^*-/-*^ mice compared to individual cytokine knockouts [22]. As expected, WT controls cleared *C*. *albicans* within 5 days, while mice lacking IL-22, IL-22RA1, or IL-17RA had high fungal burdens (approximately 10^3^ CFU/g) and weight loss (5% to 10%) (Fig 1A and 1B) [23]. Strikingly, *Il22ra1Il17ra*^*-/-*^ mice exhibited approximately 1 log elevated fungal loads and more weight loss compared to individual knockouts (Fig 1A and 1B). *Il22ra1Il17ra*^*-/-*^ mice experienced severe morbidity, requiring sacrifice by days 8 to 10, which is rare in individual knockouts (Fig 1C) [23].

Esophageal fungal loads were higher in *Il22ra1Il17ra*^*-/-*^ mice compared to *Il22ra*^*-/-*^ mice, but were not statistically different from *Il17ra*^*-/-*^ animals (Fig 1D). *Il22ra1Il17ra*^*-/-*^ mice showed reduced colon lengths (Fig 1E), indicative of intestinal tissue damage. However, fungal loads in the SI and colon were the same in all mouse strains (Fig 1D), collectively suggesting that the oral cavity shows a particular sensitivity to combined actions of these cytokines. There was no fungal dissemination to visceral organs (kidney, liver, and spleen) (Fig 1F), likely ruling out systemic candidiasis as cause of mortality. Thus, IL-17 and IL-22 act cooperatively to limit oral and to some extent esophageal candidiasis, but not intestinal colonization of this fungus.

### The SPRR family is implicated in oral candidiasis

**Fig 2:**
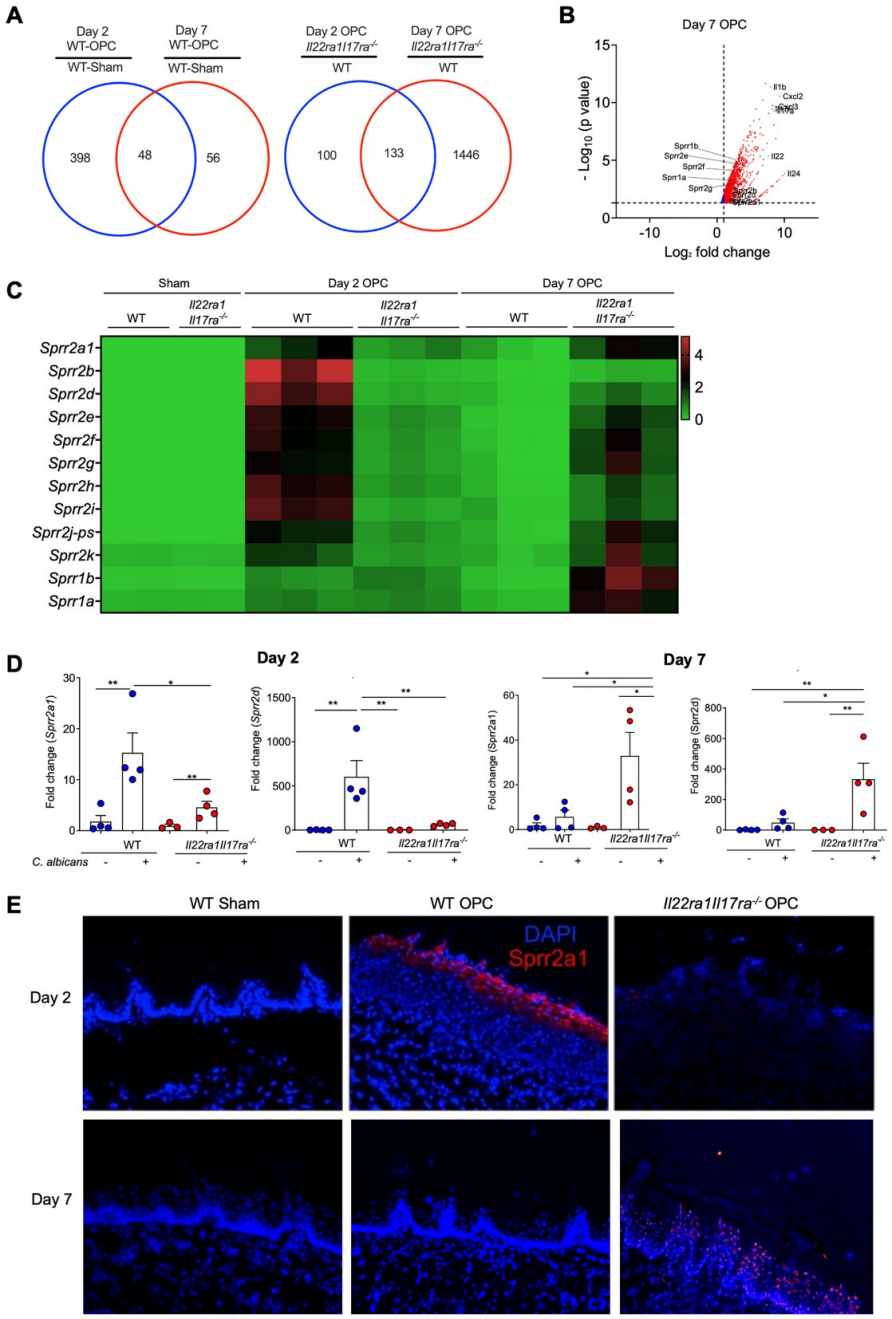
*IL-22R/IL-17R* deficiency implicates SPRRs. **(A)** Illumina RNA-Seq analysis of tongue mRNA. **(B)** Transcriptional changes in OPC. Gene expression was normalized between *Il22ra1Il17ra*^*-/-*^ and WT subjected to OPC at day 7. **(C)** Heatmap of selected genes normalized to sham. **(D)** Gene expression by qPCR normalized to *Gapdh*. Data show ± SEM relative to WT untreated mice, ANOVA with Tukey’s test. **(E)** Tongue cryosections at day 5 p.i. were stained with DAPI and anti-Sprr2a1 Abs. Representative of 3–5 mice/group.

To understand how IL-22 and IL-17 act in OPC, we compared oral mRNA profiles after OPC in WT versus *Il22ra1Il17ra*^*-/-*^ mice, revealing unique and overlapping expression patterns (Fig 2A). We focused on gene changes in *Il22ra1Il17ra*^*-/-*^ mice immediately prior to severe morbidity (day 7), where 1,446 genes were expressed in *Il22ra1Il17ra*^*-/-*^ compared to WT controls (Fig 2B). A gene set not previously linked to antifungal immunity encoded antimicrobial small proline-rich proteins (SPRRs) (Fig 2C and 2D) [21,24,25]. *Sprr* expression was negligible at baseline but induced in WT mice at early time points (day 2). By day 7 when *C*. *albicans* was cleared, SPRRs were no longer evident. In *Il22ra1Il17ra*^*-/-*^ mice, SPRRs were impaired at day 2 though up-regulated at day 7, commensurate with high fungal burdens. IF staining confirmed oral expression of a representative SPRR, Sprr2a1, in WT mice at day 2 but not *Il22ra1Il17ra*^*-/-*^ mice (Fig 2E).

### SPRR2a mediates anti-Candida responses

**Fig 3:**
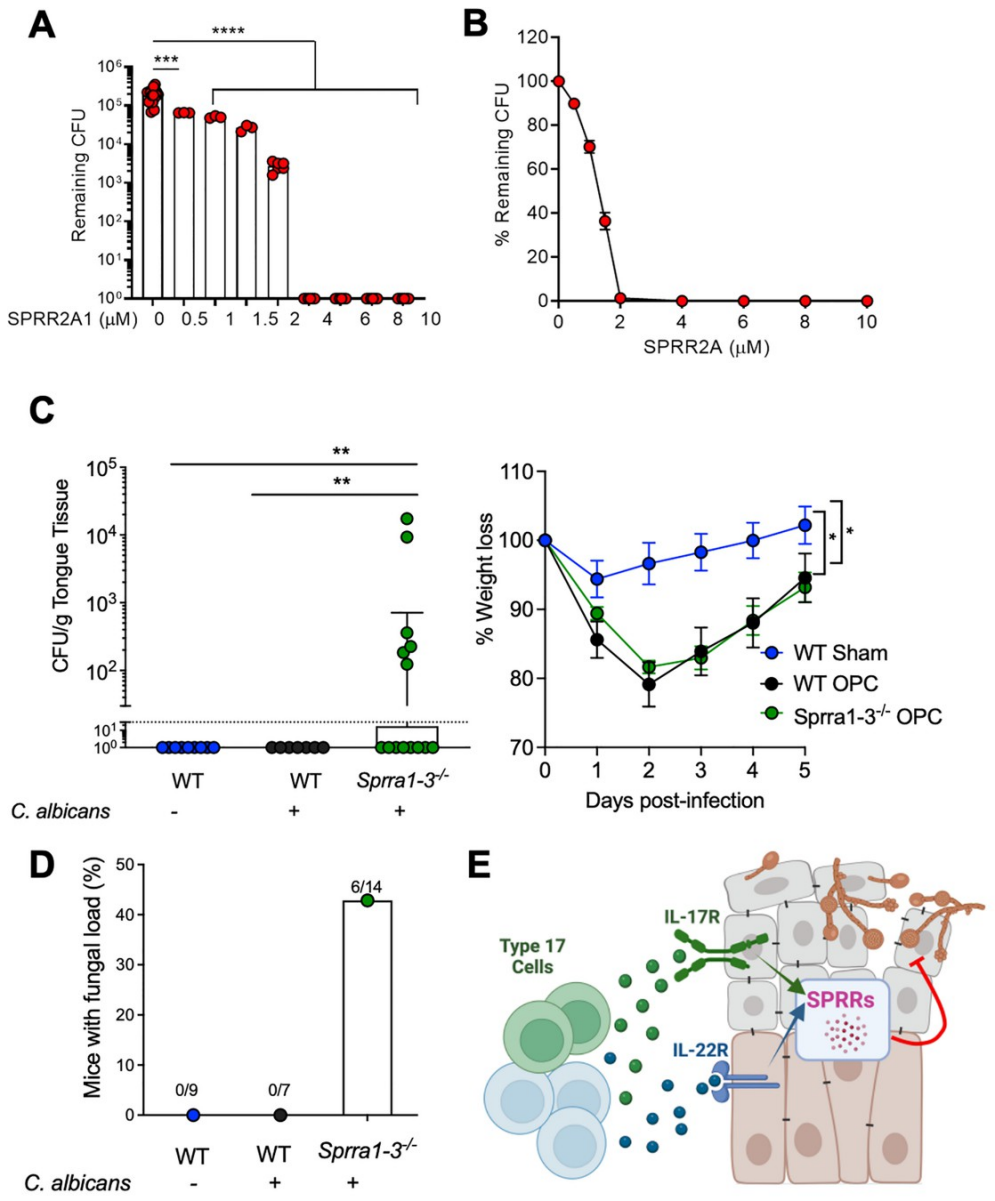
Sprr2a controls *C*. *albicans*. **(A, B)** *C*. *albicans* were cultured with Sprr2a (0.5–10 μm) for 2 h and CFU enumerated. Two-way ANOVA with Tukey’s test. **(C)** Left: fungal loads on day 2 by ANOVA with Dunn’s test. Right: weight loss. Analyzed by two-way ANOVA. **(D)** Percent of mice with fungal loads/cohort. Data pooled from 2 independent experiments. **(E)** Model of IL-17/IL-22 cooperative signaling in the oral mucosa. Created with Biorender.com.

To ascertain whether SPRRs have antifungal properties, *C*. *albicans* cells were cultured in vitro with recombinant SPRR2A1 and surviving fungi enumerated. There was a dose-dependent candidacidal effect of Sprr2A1 (Fig 3A and 3B). In vivo, mice lacking 3 Sprr2a genes (*Sprr2a1*, *Sprr2a2*, and *Sprr2a3*) were significantly but modestly susceptible to OPC, showing elevated fungal loads and increased percentages of mice infected (Fig 3C and 3D). However, OPC was not as severe in SPRR-deficient mice as in IL-17RA or IL-22RA knockouts, given the low fungal loads and full recovery from infection-induced weight loss (Fig 3C). Thus, SPRR family members are up-regulated in OPC and exhibit direct antifungal activity (Fig 3E), though clearly additional antifungal pathways are also operative.

## Discussion

These data show that IL-22R/IL-17R deficiency causes severe susceptibility to OPC, far more than loss of either cytokine receptor alone (Fig 3). Synergistic activities of IL-17 and IL-22 on target cells (typically nonhematopoietic) has been described previously, and particularly relevant here is cooperative up-regulation of antimicrobial effectors (β-defensins, S100 proteins) in dermal keratinocytes [26–28]. The present signaling synergy was so profound that *Il22ra1Il17ra*^*-/-*^ mice subjected to OPC required sacrifice, which was almost never seen in individual knockouts [17,23]. Although the basis for their mortality is uncertain, likely contributing factors are severe oropharyngeal/esophageal inflammation that impairs nutritional intake (evidenced by weight loss), and potentially fatal dissemination of intestinal bacteria, described recently by Drummond and colleagues [29].

SPRRs are best understood as precursors for the cornified envelope in stratified squamous epithelia [21,30], prominently expressed in conditions of hyperkeratinization and dermal inflammation [31], but are now appreciated to have important antimicrobial activities. For example, mice lacking *Sprr1a* and *Sprr2a* show increased susceptibility to MRSA and dermal *P*. *aeruginosa* infections [32]. SPRR2A causes membrane disruption in several bacterial species, many of which reside in the oral cavity, and also limits bacterial adherence to gastrointestinal epithelium [21,32]. A recent report demonstrated anti-helminth properties of SPRRs [25]. We show that at least 1 family member (SPRR2A) exhibits direct anti-*Candida* activities in vitro, and SPRRs contribute to control of candidiasis in vivo. Although expression of SPRRs at early time points (day 2) was strongly IL-17/IL-22-dependent, evidently other inflammatory events associated with high fungal loads drive expression at later times (day 7). To our knowledge, this is the first connection of SPRRs to fungal host defense, and future studies may determine whether direct application of SPRRs to the oral mucosa can be exploited as an antifungal therapy.

IL-17 and IL-22 are produced by “Type 17” lymphocytes (Th17 cells, γδ T cells, ILC3s), but their downstream mechanisms of action are distinct [7,19]. IL-17 signaling is mediated by proximal adaptors (Act1, TRAF6), which orchestrate an inflammatory signaling through NF-κB, MAPK, C/EBPs, and posttranscriptional pathways that stabilize inflammatory mRNA transcripts [33]. IL-22, in contrast, drives a JAK kinase-STAT3 pathway, controlling genes involved in tissue repair, wound healing, and proliferation [34]. In this regard, STAT3 up-regulates SPRRs that modify the epithelial barrier under gastrointestinal stress [32,35], though relatively little is known about how SPRRs are regulated.

Our assessment of SPRR2a (one of the few SPRRs for which detection reagents are available), showed prominent expression in the oral epithelium during OPC. Oral epithelial cells (OECs) are key responders to Type 17 cytokines, yet there is a clear separation of cytokine-responsive cell types within this tissue [17,36,37]. Specifically, while IL-17 signals in the superficial, K13^+^ post-mitotic OEC layer via the IκBζ transcription factor [23,36], IL-22 is required mainly on the proliferating K14^+^ basal stem-like cell layer [17,36]. Admittedly, this view of oral epithelial cell dynamics is overly simplistic, as cells in the basal layer undergo complex transitional states during differentiation [38]. More in-depth characterization of how and where antifungal effectors such as SPRRs are regulated during candidiasis is warranted.

In summary, SPRRs, in part regulated by Type 17 pathway cytokines, make clear contributions to oral antifungal defense. Whether SPRRs play roles in other forms of candidiasis remains to be determined. These observations may have clinical utility, given the emergence of multidrug-resistant fungal strains and the unmet need for strategies to target such infections [1,5].

## Methods

### Ethics statement

All animal studies were performed with approval from the University of Pittsburgh Institutional Animal Care and Use Committee under protocol # 23083550 and were compliant with all applicable provisions established by the Animal Welfare Act and the Public Health Services (PHS) Policy on the Humane Care and Use of Laboratory Animals.

### Mice

*Il22ra1*^*fl/fl*^ [39] were crossed to *Il17ra*^*-/-*^ (Amgen, Thousand Oaks, California). *Il22*^*-/-*^ were from Genentech, *Sprra1-3*^-/-^ from JAX, WT C57BL/6 mice from JAX or Taconic. Experiments were performed on age-matched mice (6 to 10 weeks, both sexes) housed in SPF conditions.

### OPC

OPC was induced by sublingual inoculation with 10^7^ CFU *C*. *albicans* (CAF2-1) or PBS (Sham) in saturated cotton balls for 75 min [22,23]. Tissue homogenates were prepared on a GentleMACS Dissociator (Miltenyi Biotec) with C-tubes. Intestinal tissue was flushed with PBS prior to homogenization. CFU was determined by serial dilution plating on YPD/Amp.

### Candidacidal assessment

*C*. *albicans* (CAF2-1) cells (10^5^ cells/ml) in serum-free RPMI were cultured with rSPRR2A (MBS1345074) for 2 h and CFU assessed.

### Immunofluorescence (IF)

Cryosections were stained with DAPI and anti-SPRR2A (MBS9209523) and goat anti-mouse AF488 (A32723, Invitrogen). Images were acquired on an EVOS FL microscope.

### qPCR and RNASeq

Tongue RNA extracted using RNeasy kits (Qiagen). Primers were from Quantitect. Nextera XT RNA sequencing was performed on Illumina NextSeq 500 and analyzed by CLC Genomics Workbench v. 22. QC was performed on FASTQ RNASeq reads. Reads with Phred score >20 were aligned to a reference genome (mm10, GRCm38.75) with default parameters. DEGs were filtered for significance at log fold change (LFC) ≥ 1, and *p* ≤ 2. G profile analysis was conducted using default parameters. Data are available at the Sequence Read Archive http://www.ncbi.nlm.nih.gov/bioproject/1094974.

### Statistics

Significance determined by ANOVA and indicated post hoc multiple comparison tests, analyzed on GraphPad Prism. *P* < 0.05 considered significant. *P < 0.05, ** <0.01, ***<0.001, ****<0.0001.
